# Form and Motion

**Published:** 1870-04

**Authors:** Z. C. McElroy

**Affiliations:** Zanesville, Ohio


### Correspondence.

### "FORM AND MOTION."

ZANESVILLE, OHIO, *March* 2, 1870.

*To the Editors of* THE CHICAGO MEDICAL JOURNAL:

PLEASE convey to your critical correspondent* my thanks for calling attention to my communication,† in which form and motion were pointed out as the physiological and physical unities of organic life. Say to him he may hew away at his, not mine, ideals of "figure" and "moving about," to his heart's content, and I shall never say, stay thy hand.

As he said nothing for or against form and motion as I used

* CHICAGO MEDICAL JOURNAL, *March*, 1870, p. 134.

† CHICAGO MEDICAL JOURNAL, *January*, 1870, p. 6.

them, no further reply than this acknowledgment of my thanks for his courtesy is necessary.

To reproduce in other minds the exact impressions of our own, by the arbitrary symbols of language, has ever been environed with difficulties.* The tiresome repetitions in legal documents are due to this cause, though other illustrations are neither far to seek nor difficult to find. To avoid this in science, new words are coined to express different shades of meaning from those in common use; though these differences have to be pointed out in common words at last; and the use of new or unusual words, where no pressing necessity for such exists, savors of pedantry.

In my case this seemed unnecessary, as "form and molecnlar motion," as commonly understood, appeared to be sufficiently definite, especially as they were illustrated by actual clinical cases.

Since the publication of my memoir in THE JOURNAL, the following remarks on form and motion I find in an apparently recently published work,† as there used. The author does not, however, claim for them the importance to which my investigations have led me, nor did they meet my eye until after the appearance of my memoir in your pages. They, perhaps, are better than my own, because they, apparently, are calculated to reproduce in the minds of others my own mental impressions, which are here represented with a good deal of exactness.

"True idea of form." "We may repeat here that in the true idea of the Form of an object is involved not merely its *structure*, or that part of its nature which the anatomist is concerned with; it includes also the whole of the qualities and dispositions which pertain to it, and which distinguish it *socially* from other things. And this, in fact, is its *essential* nature, being that which gives it a place and a function in the general economy of creation; thus the object and end for which it was created. The end is always nobler than the Means, for the means are only processes by which the end shall be attained. In all our groupings and classifications, therefore, we should view the organic structure as

* *Atlantic Monthly*, October, 1864.

† *Life; its Nature, Varieties and Phenomena*; by Leo. H. Grindon; 5th American edition; 1869.

intermediate between the Artist and the end He has in view." Page 495.

" Reviewing these various and wonderful processes, we cannot fail to observe how, in its every phase and expression, the great sign and certificate of life is MOTION. Usefully, then, may we pause upon the consideration of it as a kind of summary and continent of vital phenomena. Nothing exists independently of motion as its cause; by reason, likewise, of motion, all things hold together and preserve their form. 'Passive life,' sometimes spoken of, is a contradiction in terms; certain states of being may be relatively passive, but there is no such thing as absolute passivity. In no case a state *ipso facto*, passivity is everywhere an incident of motion, consequently to be referred to motion, and to be explained by motion. Doubtless there is a great diversity in the degree and amount of motion; also in its manifestation to the eye. We must not confound it with *moving about*. Motion, ordinarily so called, implying visible change of place and position, and furnishing us with ideas of time, does not comprise the All of motion. There is motion which no eye can perceive, motion which we are made aware of only by witnessing its results. Of this kind, indeed, is the chief part; the most wonderful and efficient movements in the world are those which proceed in secrecy and silence. The feebler and briefer the exhibition of motion, especially the latter, the lower is the expression of life; the more energetic and continuous it is, the higher is the life—so apart from structure, motion is a criterion of vital excellence, of course under the reservation that the quality of life depends primarily and essentially on its End; else would the sea be more living than a plant; and a watch, or other piece of self-acting mechanism, commend itself as of nobler nature than many animals." Page 100–101.

If agreeable to you, further illustrations, based on clinical cases, will be prepared for your pages during the present year.

Z. C. McELROY, M.D.

